# Curcumin alleviates rheumatoid arthritis progression through the phosphatidylinositol 3-kinase/protein kinase B pathway: an *in vitro* and *in vivo* study

**DOI:** 10.1080/21655979.2022.2078942

**Published:** 2022-05-24

**Authors:** Zihan Xu, Wei Shang, Zhiming Zhao, Beibei Zhang, Chunli Liu, Hui Cai

**Affiliations:** a The First Clinical Medical College, Nanjing University of Chinese Medicine, Nanjing, Jiangsu, China; b Department of Integrated Traditional and Western Medicine, Jinling Hospital, School of Medicine, Nanjing University, Nanjing, Jiangsu, China

**Keywords:** Curcumin, rheumatoid arthritis, TNF-α, collagen-induced arthritis, fibroblast-like synoviocytes

## Abstract

Rheumatoid arthritis (RA) is a chronic, systemic autoimmune disease characterized by synovial inflammation and joint bone and cartilage destruction. Curcumin can improve joint inflammation in rats with arthritis and inhibit synovial revascularization and abnormal proliferation of fibroblasts. However, it is unclear whether curcumin affects the RA progression. The TNF-α-stimulated primary RA fibroblast-like synoviocytes (RA-FLS) and SV-40 transformed MH7A cells were used as the *in vitro* model of RA. A mouse model of collagen-induced arthritis (CIA) was used as the *in vivo* model. The effects of curcumin on cell proliferation, apoptosis, migration, invasion, and inflammatory response were assessed by colony formation, flow cytometry, wound scratch, Transwell assays, and western blotting analysis. Arthritis index scores and degree of paw swelling in mice were assessed to evaluate RA. Curcumin inhibited the TNF-α-induced proliferation, migration, invasion of MH7A and RA-FLS cells and promoted cell apoptosis. Administration with curcumin reversed the CIA-induced increase in arthritis scores, hind paw edema, and loss of appetite, while these effects were rescued by insulin-like growth factor 1, the upstream cytokine of PI3K/AKT. Moreover, curcumin suppressed the inflammatory response by reducing TNF-α, IL-6, and IL-17 secretion in CIA-stimulated mice. Curcumin has an excellent anti-RA effect *in vivo* and *in vitro*, which is exerted by inhibiting the expression of pro-inflammatory factors TNF-a, IL-6 and IL-17 and inhibiting the activation of PI3K/AKT signaling pathway. Thus, curcumin may be a promising candidate for anti-RA treatment.

## Graphical Abstract

**Figure:**
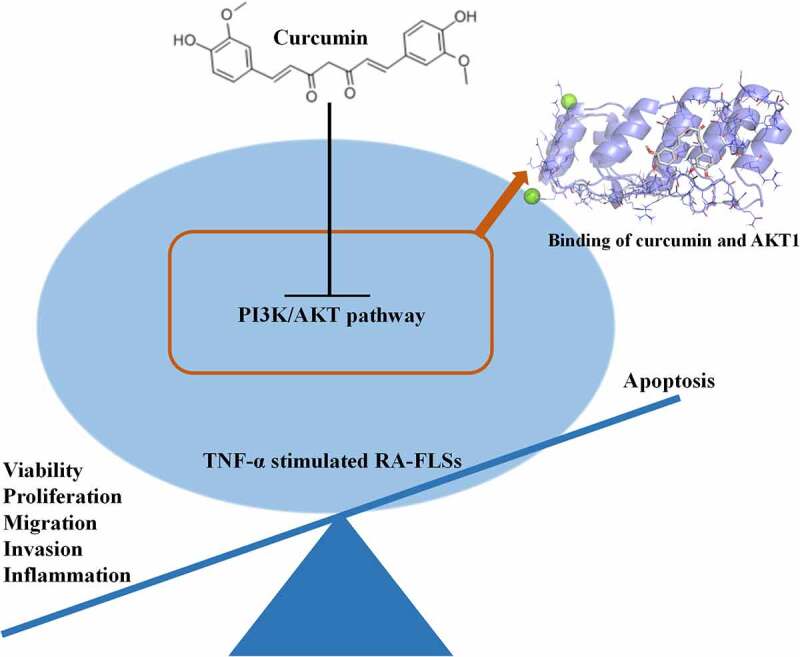

## Highlights

- Curcumin suppresses cell and promotes apoptosis in RA-FLS.
- Curcumin reduces cell migration, invasion, and inflammatory response in RA-FLS.
- Curcumin inactivates the PI3K/AKT pathway.
- Curcumin alleviates CIA in mice.

## 1. Introduction

Rheumatoid arthritis (RA) is a chronic inflammatory disease that primarily occurs in the joints and can cause damage to cartilage and bone, even leading to disability [1]. The incidence of systemic osteoporosis is related to the severity of RA arthritis, and patients with RA are at increased risk of osteoporotic fractures [2]. The pathological manifestations of RA are synovial inflammation, vascular hyperplasia (vascular opacification), immune cell infiltration, and progressive joint destruction [3]. The abnormal proliferation, apoptosis, migration, and invasion of fibroblast-like synoviocytes (FLSs) are the characteristic hallmarks of RA [4]. As the disease progresses, immune cell infiltration occurs in the joint, activating RA-FLS and promoting inflammatory factors such as interleukin −6 (IL-6) and tumor necrosis factor alpha (TNF-a) [5]. Up to date, there is no curative treatment for RA worldwide. It is often treated clinically with non-steroidal anti-inflammatory drugs, disease-modifying anti-rheumatic drugs, glucocorticoids, botanicals, and biologics [6]. Most of these drugs are used to suppress the inflammatory response and relieve the symptoms of RA, with a variety of side effects. Therefore, to effectively prevent or alleviate the progression of RA disease has become the focus of clinical attention.

*Curcuma longa*, a member of the Zingiberaceae, has rhizomes below the ground [7]. Curcumin is a tetraterpenoid obtained from *Curcuma longa*, and its anti-inflammatory, antioxidant, and anti-proliferative activities have been widely reported [8–10]. A clinical study by Chandran *et al*. evaluated the safety of curcumin and found that it could improve the outcomes of RA patients [11]. Kloesch *et al*. showed that curcumin has strong anti-inflammatory properties and induces apoptosis in human FLSs, suggesting that curcumin can be used for therapy of chronic inflammatory diseases such as RA [12]. In our preliminary experimental study, curcumin improved inflammation and inhibited synovial vascular neovascularization and abnormal proliferation of fibroblasts in arthritic rats. *In vitro* experiments revealed that curcumin inhibited osteoclastogenesis. However, it is unclear whether curcumin affects the progression of RA.

The phosphatidylinositol 3-kinase (PI3K)/protein kinase B (AKT) pathway is widely found in biological cells and plays a vital role in a series of physiological and pathological activities. PI3K, a member of the phosphatidylinositol family, has the primary function of activating other substances and generating second messengers [13]. The second messenger further activates other downstream molecules, which in turn trigger a series of chain reactions. AKT, one of the downstream targets of PI3K, is involved in a series of processes such as cell growth, proliferation, protein synthesis, and transcriptional regulation [14]. Previous studies verified that the PI3K/AKT signaling pathway is abnormally activated in RA patients’ synovial tissues [15,16].

This study was designed into two parts to investigate whether curcumin influences RA at the preclinical levels. In the first part, cellular experiments were performed by stimulating MH7A and RA-FLS cells with TNF-α. After treatment with curcumin or curcumin + 740 Y-P, the agonist for PI3K/AKT pathway, the proliferation, apoptosis, migration, invasion, secretion of inflammatory factors, and PI3K/AKT pathway-associated proteins in MH7A and RA-FLS cell were observed. Second, after establishing a mouse model of RA, the effects of curcumin and curcumin + insulin-like growth factor 1 (IGF-1), the upstream cytokine of PI3K/AKT, on collagen-induced arthritis (CIA) were assessed by evaluating arthritis index scores and degree of paw swelling.

## 2. Materials and methods

### 2.1. Construction of curcumin-rheumatoid arthritis interaction network

The 2D structure of curcumin was downloaded from PubChem (https://pubchem.ncbi.nlm.nih.gov/). The Swisstarget database (http://swisstargetprediction.ch/) was used for predicting curcumin targets. From the GeneCards database (https://www.genecards.org/), ‘Rheumatoid Arthritis’ was used as a keyword to collect RA disease targets. The intersection of the targets of curcumin and RA was presented by the Venn diagram. A drug–disease interaction network was constructed. The string online database (https://cn.string-db.org/) was used to construct the protein interaction network for the targets of curcumin in the treatment of RA. Cytoscape 3.7.2 software was used to screen out the core targets of curcumin in the treatment of RA. AKT1 crystal structure was obtained from RCSB Protein Data Bank (https://www.rcsb.org/) with the PDB ID of 5AAR. Relative parameters were listed as follows: Method: X-RAY DIFFRACTION; Resolution: 1.87 Å; R-Value Free: 0.253; R-Value Work: 0.197; R-Value Observed: 0.200. Autodock 4.2 was used for hydrogenation and dewatering pretreatment of the AKT1 structure. PyMoL and AutoDock softwares were used to preprocess, dock, and visualize proteins and compounds. GO (https://david.ncifcrf.gov/) and KEGG (https://www.genome.jp/kegg/) enrichment analysis was performed to investigate the biological functions of the targets and the association analysis of the involved pathways.

### 2.2. Cell culture and curcumin treatment

Human rheumatoid fibroblast-like synoviocyte transformed with SV40 T antigen, MH7A (#MZ-0651; Mingzhou Bio; Ningbo, China), and primary human RA-FLS (#TOPCP0002; TOPBIOTECH, Shenzhen, China) were used for this study. All cells were cultured in DMEM containing 1% antibiotics (100 U/mL penicillin, 100 U/mL streptomycin) and 10% fetal bovine serum at 37°C and 5% CO_2_. The cells were observed microscopically when they grew to 80%–90% confluency, and subsequent experiments were performed.

TNF-α (5, 10, 20, 50 ng/mL) was used to treat MH7A or RA-FLS cells for 24 h to mimic the *in vitro* condition of RA. For curcumin treatment, different doses of curcumin (10, 25, 50, and 100 μM) were added in the medium for 12, 24, 48 h, and 72 h of incubation. In some experiments, cells were cotreated with 50 μM curcumin and 10 ng/mL IGF-1 or 20 μg/mL 740 Y-P for 48 h to activate the PI3K pathway.

### 2.3. MTT assay

RA-FLS and MH7A cells were seeded into 96-well plates at a density of 8000 cells/well. Next, 10 μl of MTT reagent was added to each well and incubated at 37°C with 5% CO_2_ for 4 h. Afterward, 100 μL of DMSO was added to each well followed by shaking for 10 min to fully dissolve the crystals. Finally, the OD value was measured at 570 nm using a universal microplate spectrophotometer.

### 2.4. 5-Ethynyl-2’-deoxyuridine (EdU) assay

Cells in logarithmic growth phase were inoculated in 6-well plates at the density of 1 × 10^5^ cells/well. About 100 μL of EdU medium was added to each well, and cells were incubated with the EdU medium for 2 h. Cells were fixed with 4% paraformaldehyde for 15 min and then centrifuged at 1000 rpm for 5 min. About 500 μL of Apollo8 staining reaction solution was added to resuspend the cells, followed by incubation for 30 min at room temperature and centrifugation at 1000 rpm for 5 min. Next, cells were permeabilized by 500 μL of 0.5% TritonX-100 in PBS, centrifuged at 1000 rpm for 5 min, and washed with 100 μL of methanol 1–2 times for 5 min. About 500 μL of Hoechst 33,342 reaction solution was added, and cells were incubated with the Hoechst 33,342 reaction solution for 30 min at room temperature. Finally, cells were washed by PBS sand centrifuged at 1000 rpm for 5 min three times to elute Hoechst 33,342 reaction solution.

### 2.5. Colony formation assay

The cells of each group in the logarithmic growth phase were digested with 0.25% trypsin, blown into individual cells and suspended in 10% FBS in the DMEM culture medium. The cell suspension was diluted and inoculated into 10 mL of 37°C pre-warmed culture medium at a gradient density of 50, 100, and 200 cells/dish. All cells were incubated at 37°C under a condition of 5% CO_2_ and 90% humidity for 2 ~ 3 weeks. The culture was terminated when clones appeared in the dishes. After removing the supernatant and washing carefully with PBS two times, 5 mL of 4% paraformaldehyde were added to fix cells for 15 minutes. The fixation solution was removed, and cells were cultured with the GIEMSA staining solution for 10 ~ 30 min. The dish was inverted, and the transparent film was superimposed with a grid. Finally, clones with more than 50 cells were counted under the microscope.

### 2.6. Flow cytometry apoptosis analysis

The treated cells were inoculated in six-well plates at a density of 1 × 10^6^/well and incubated at 37°C and 5% CO_2_. Twenty-four hours later, 5 uL of Annexin V and 5 uL of PI were added for 15 min of incubation at room temperature, avoiding light. Finally, the cells were detected on a flow cytometer on a BD FACSCalibur system.

### 2.7. Wound healing assay

Treated cells were incubated in 96-well plates with a density of 2 × 10^4^/well at 37°C when cell reached 80%–90% confluency. A 100 μL sterile tip was used to gently scratch the cell monolayer to generate a straight line with a suitable width. After washing with PBS two times, the medium without the serum was added. Five randomly selected fields of view were photographed and marked under a microscope at a magnification of 100 × . Afterward, the cells were incubated in a 37°C incubator for 24 h. The same fields were photographed under the microscope according to the markings and photographed at a magnification of 100 ×. Scratch width calculation was performed using the ImageJ software.

### 2.8. Transwell assay

Matrigel was diluted with DMEM, and 100 uL of Matrigel was applied to the surface of the Transwell membrane. The Matrigel-coated chambers were placed in a 37°C incubator for 30 min. Cells were inoculated in a 24-well plate at a density of 3 × 10^4^/mL, and 500 ul of complete medium containing 20% FBS was added. The cells were incubated in the incubator at 37°C for 24 h. Afterward, the cells in each well were stained with 1 mL of 0.01% crystal violet solution for 20 min. Five randomly selected fields of view were photographed under the microscope, and the average number of cells was recorded.

### 2.9. Western blotting

Total proteins from cells and tissues were extracted by the RIPA lysate. Protein quantification was conducted with a BCA protein quantification kit. An appropriate amount of protein sample was denatured at 100°C for 3–5 min and then subjected to protein electrophoresis using 10% SDS-PAGE for 1 h. the samples were transferred to PVDF membranes. The membranes were blocked in Tris Buffered Saline with Tween 20 blocking solution containing 5% skim milk powder at room temperature for 2 h. The membranes were washed three times with TBST and incubated overnight at 4°C using primary antibodies against MMP-2, MMP-9, p-PI3K, PI3K, p-AKT, AKT, and GAPDH (ab92536, ab76003, ab278545, ab140307, ab192623, ab8805, ab8245; all from Abcam, Shanghai, China). The next day, the horseradish peroxidase-labeled goat anti-rabbit secondary antibody IgG (ab96899) and goat anti-mouse secondary antibody IgG (ab96879) dilution buffer were added, and membranes were incubated with these secondary antibodies at room temperature for 2 h. The enhanced chemiluminescence reagent was added dropwise, and the membranes were imaged by the gel imaging system. The gray value of each band was analyzed by the ImageJ software. The results were expressed as the ratio of the gray value of the target protein to GAPDH, the internal reference protein.

### 2.10. Quantitative real time polymerase chain reaction (qRT-PCR) analysis

Total RNA was extracted from cells using TRIzol reagent (Invitrogen, USA) as per the manufacturer’s protocols. A PrimeScript^TM^ RT Reagent Kit (Takara, Japan) was applied to synthesize cDNA from RNA following the manufacturer’s instructions. Afterward, the PCR reaction was conducted using SYBR Green Realtime PCR Master Mix Reagent (Takara, Japan) on an ABI 7500 system according to the manufacturer’s instructions. The PCR amplification procedures included initial denaturation at 94°C for 5 min, 40 cycles of 94°C for 5 s and 62°C for 30 s. The primers sequences were as follows: TNF-α, forward, 5'-GAGTGACAAGCCTGTAGCCCATGTTGTAGC-3', and reverse, 5'-GCAATGATCCCAAAGTAGACCTGCCCAGACT-3'; IL-6, forward, 5'-ATGAACTCCTTCTCCACAAGCGC-3', and reverse, 5'-GAAGAGCCCTCAGGCTGGACTG'-3'; IL-17 forward, 5'-CGATGACTCCTGGGAAGACCTC-3', and reverse, 5'-GTGTGGGCTCCCCAGAGCTCTTA-3'; GAPDH forward, 5'-TGAAGGTCGGAGTCAACGGATTTGGT-3', and reverse, 5'-CATGTGGGCCATGAGGTCCACCAC-3'.

### 2.11. Construction of a mouse model of RA

The animal experiments were performed under the approval of the Animal Ethical Committee of Jinling Hospital, School of Medicine, Nanjing University (2021JHDWLS-002). All experimental mice were acclimatized and fed for 7 days before modeling. Male DBA/1 mice were randomly divided into four groups: control group, CIA group, CIA+curcumin group, and CIA+curcumin+IGF-1 group, with eight mice in each group. Under aseptic and light-proof conditions, type II collagen (C II) powder (Chondrex, Inc., Redmond, WA, USA) was dissolved in 0.02 mol/L acetic acid solution to a final concentration of 2 mg/ml and then stirred well in a refrigerator at 4°C overnight. On the second day, under the same conditions, C II solution was emulsified with an equal volume of CFA solution (Chondrex, Inc.) at the concentration of 4 mg/ml on ice to form a white emulsion. The suspension (0.15 mL) was injected intradermally into the tail root of mice at a dose of 200 μg CII/animal. Fourteen days later, the suspension was injected intradermally into the tail root and three points on the back at a dose of 0.1 ml/animal and 100 μg CII/animal, respectively, to intensify the stimulation of immunity. After the intensified immunization, all mice showed increased levels of skin rupture, redness, and swelling of the hindfoot at the injection site. Some mice showed deformation of the toes of the hindfoot and crawled slowly, often with the paws upward without touching the bottom of the cage. The control group was injected with an equal amount of saline at the same location.

The intervention group was treated with curcumin on day 21 of modeling. The CIA+curcumin group was given 50 mg/kg of curcumin (MedChemExpress, Princeton, NJ, USA) dissolved in 0.5% sodium carboxymethylcellulose (CMC-Na) by gavage once a day for 28 days. Mice in the control or CIA group also received the same dose of curcumin solvent CMC-Na by gavage once a day for 28 days to exclude its side effects. The CIA+curcumin+IGF-1 group was given 50 mg/kg of curcumin [17,18] dissolved in 0.5% CMC-Na by gavage once a day for 28 days and an intravenous injection of 2 mg/kg recombinant mouse IGF-1 (R&D systems).

At the end of the experiment, the mice were anesthetized by intraperitoneal injection of 10% chloral hydrate (0.3 mL/100 g). The synovial tissues were harvested for following biochemical assays.

### 2.12. Enzyme-linked immunosorbent assay (ELISA)

The levels of TNF-α, IL-6 and IL-17 in mice serum samples and RA-FLSs were detected by ELISA. The corresponding ELISA kits (TNF-α, kt99985; IL-6, BA23048; IL-17, kt22800) were purchased from MSK (Wuhan, China).

### 2.13. Scoring of arthritis index

The degree of RA in the mice was observed and recorded after immunization every 7 days. The arthritis index was calculated by accumulating the points of the degree of lesions of the two hind ankle joints with reference to the 4-point scale evaluation: 0 points: no obvious abnormality; 1 point: mild swelling of the small toe joint; 2 points: moderate swelling of the toe joint and the plantar area of the foot, 3 points: swelling of the paw below the ankle joint; 4 points: swelling or even deformity of all joints including the ankle joint.

### 2.14. Paw swelling evaluation

During the experiment, the thickness of bilateral hind paws was measured using a vernier caliper every seven days. Rate of paw swelling change (%) was defined as follows: [paw thickness on day 49 after immunization] – [paw thickness on day 0 prior immunization]/[paw thickness on day 0 prior to immunization] × 100%.

### 2.15. H&E staining

At the end of the experiment, the mice were euthanized. The inflammatory swollen joints were cut off to remove the excess flesh and fixed in 4% paraformaldehyde for 48 h. After decalcification with EDTA, the joints were cut longitudinally from the center and placed in the embedding box and rinsed with running water overnight. Samples were dehydrated in a concentration gradient of ethanol at room temperature, immersed in the wax, and embedded using a semi-automatic embedding machine. The slides were cut into 4 μm sections using a microtome, dewaxed in xylene, and sequentially rinsed from ethanol to water. The sections were then stained with hematoxylin for 10 min, fractionated in 0.5% hydrochloric acid ethanol for 30 ~ 60 s, washed with low speed running water for 5 min, followed by treatment with 80% ethanol for 1 min and eosin stain for 1 min. Dehydration, transparency, and sealing were routinely performed.

### 2.16. Immunofluorescence staining

Sections from joint samples were incubated with the anti-Bax (ab32503, Abcam) primary antibody at 4°C overnight. The secondary antibody was the Rabbit Anti-Goat IgG (ab175667, Abcam). 4’,6-diamidino-2-phenylindole (DAPI) was used to stain the nuclei. An Olympus BX-51 microscope was used to photograph the images.

### 2.17. Statistical analysis

The data in the present study were expressed as mean ± SD, and SPSS 22.0 statistical software was applied for data analysis. All results were derived from more than three independent experiments. One-way analysis of variance followed by Tukey’s *post hoc* test or two-way analysis of variance was used for statistical analysis. P < 0.05 was considered to indicate statistically significant difference.

## 3. Results

### 3.1. Network pharmacological study on the mechanism of curcumin in the treatment of RA

**Figure 1.**
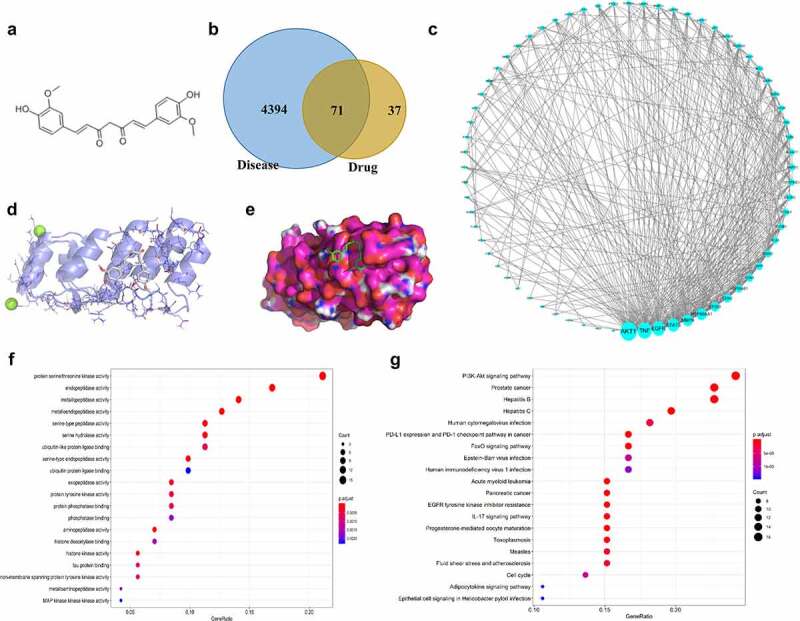
Network pharmacological study on the mechanism of curcumin in RA. (a) 2D structure of curcumin. (b) Intersection of the targets of RA and curcumin. (c) Curcumin-RA target–protein interaction network. (d) Docking diagram of curcumin and AKT1. (e) Surface image of curcumin docking with AKT1. (f) GO enrichment analysis results. (g) KEGG enrichment analysis results.

A network pharmacological study was performed to reveal the mechanism of curcumin in RA. The 2D structure of curcumin is shown in Figure 1(a). A total of 108 curcumin targets were obtained by the Swisstarget database. Through the GeneCards database, a total of 4465 targets of RA were collected. Through mutual mapping, 71 targets of curcumin for the treatment of RA were obtained (Figure 1(b)). Through the construction of core targets, a protein interaction network with AKT1 as the central core target was obtained. The main core targets were AKT1, TNF, EGFR, STAT3, and MMP9 (Figure 1(c)). The optimal affinity (kcal/mol) between PyMol and AutoDock is −5.1, indicating a good binding ability between curcumin and AKT1 (Figure 1(d,e)). As shown in Figure 1(f,g), protein serine/threonine kinase activity and PI3K-AKT signaling pathway were enriched in GO and KEGG enrichment analysis.

### 3.2. Curcumin suppressed the TNF-α-induced MH7A and RA-FLS cell proliferation, migration, and invasion and promoted apoptosis by directly regulating the PI3K/AKT pathway

**Figure 2.**
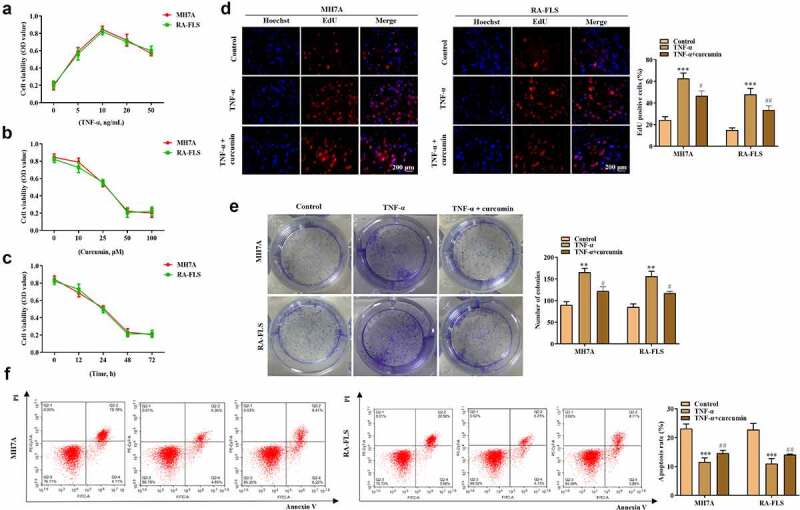
Effects of curcumin on cell viability, proliferation, and apoptosis in RA-FLSs *in vitro*. (a) MTT assay was performed to determine the effect of TNF-α in the viability of MH7A and primary RA-FLS. (b) Effects of curcumin at concentrations of 10, 25, 50, and 100 μM on the viability of MH7A and primary RA-FLS. (c) Effects of curcumin (50 μM) on cell viability of TNF-α-stimulated MH7A and primary RA-FLS after incubation for 12, 24 and 48 h. (d) EdU assay was conducted to determine MH7A and primary RA-FLS cell proliferation after treatment with 50 μM curcumin for 48 h. (e) Colony formation assay was conducted to evaluate the effect of curcumin on TNF-α-induced MH7A and primary RA-FLS cell proliferation. (f) Cell apoptosis was detected by flow cytometry analysis. **p < 0.01, ***p < 0.001 vs. control, ^#^p < 0.05, ^##^p < 0.01 vs. TNF-α.

**Figure 3.**
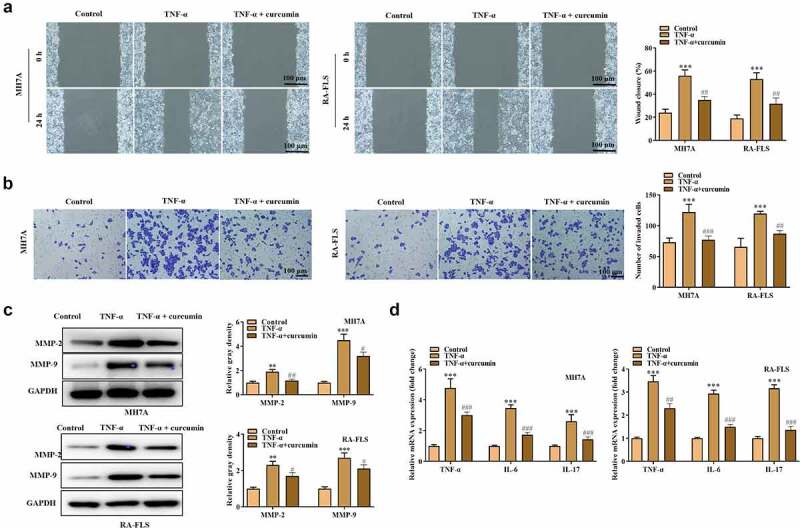
Effects of curcumin on cell migration, invasion, and inflammatory response in RA-FLS *in vitro*. (a) Wound scratch assay was used to detect cell migration. (b) Transwell assay was conducted to evaluate cell invasion. (c) Western blot analysis of MMP-2 and MMP-9 proteins in different groups. (d) Relative expression levels of inflammatory cytokines in different groups were determined by RT-qPCR analysis. **p < 0.01, ***p < 0.001 vs. control, ^#^p < 0.05, ^##^p < 0.01, ^###^p < 0.001 vs. TNF-α.

The effects of curcumin on the proliferation, migration, invasion, apoptosis, and inflammatory response of the RA-FLSs were investigated. Our preliminary experiments showed that TNF-α at the concentration of 10 ng/mL achieved best results on the viability of MH7A or RA-FLS cells (Figure 2(a)), and thus this dose was used for the following studies. Figure 2(b,c) showed that curcumin concentration- and time-dependently decreased viability of MH7A or RA-FLS. The EdU and colony formation assays in Figures 2(d,e) further demonstrated that TNF-α treatment prominently increased cell proliferation while curcumin reversed the effects of TNF-α on proliferation. Meanwhile, the flow cytometry analysis in figure 2(f) illustrated that the apoptosis in MH7A and RA-FLS cells was reduced after exposure to TNF-α. However, the reduction in apoptosis could be partially counteracted by curcumin. Moreover, the wound scratch assay, Transwell chamber assay, and western blot analysis of MMP-2 and MMP-9 proteins in Figure 3(a-c) elucidated that TNF-α treatment resulted in increased migration and invasion abilities in MH7A and RA-FLS, whereas these effects could be antagonized by curcumin. The qRT-PCR analysis in Figure 3(d) presents that TNF-α induced the inflammatory response in MH7A and RA-FLS cells. However, the TNF-α-induced increase in TNF-α, IL-6, and IL-17 levels in MH7A and RA-FLS cells could be partially counteracted by curcumin treatment.

**Figure 4.**
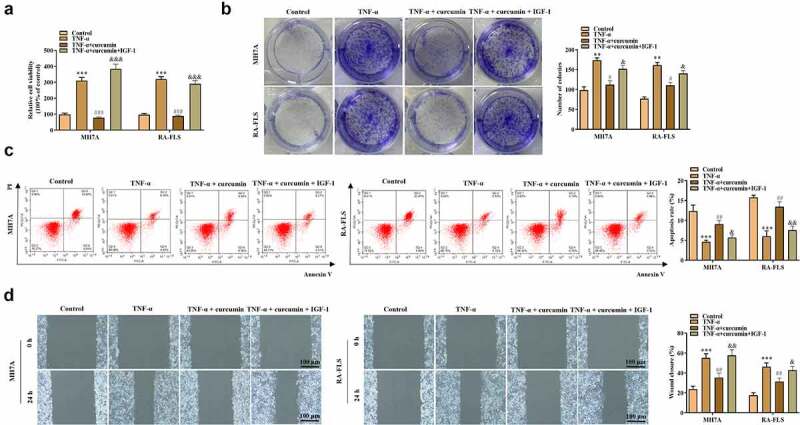
IGF-1 rescued the effects of curcumin on the viability, proliferation, migration, and apoptosis of RA-FLSs. (a-b) Cell viability and proliferation of MH7A and RA-FLS in the control, TNF-α, TNF-α + curcumin, and TNF-α + curcumin + IGF-1 groups were determined by MTT and colony formation assays. (c) Flow cytometry analysis was performed to detect cell apoptosis. (d) Cell migration was assessed by wound scratch assay. **p < 0.01, ***p < 0.001 vs. control, ^#^p < 0.05, ^##^p < 0.01, ^###^p < 0.001 vs. TNF-α, ^&^p < 0.05, ^&&^p < 0.01, ^&&&^p < 0.001 vs. TNF-α + curcumin.

**Figure 5.**
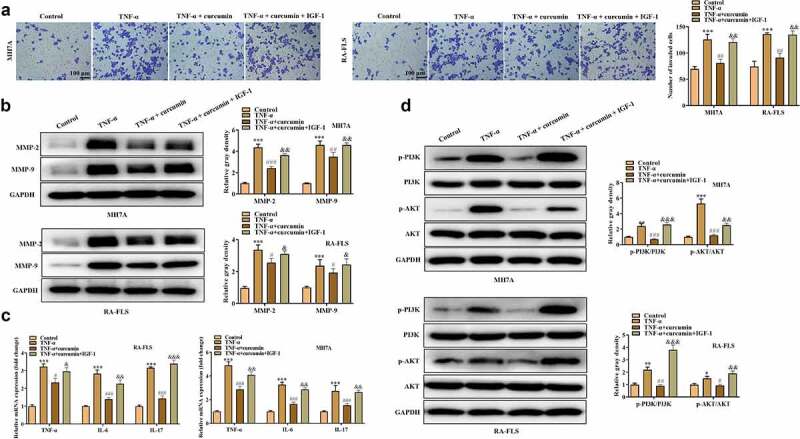
IGF-1 rescued the effects of curcumin on the migration and inflammatory response of RA-FLSs. (a) Invasion of MH7A and RA-FLS in the control, TNF-α, TNF-α + curcumin, and TNF-α + curcumin + IGF-1 groups was evaluated by Transwell assay. (b) Western blot analysis was conducted to detect protein levels of MMP-2 and MMP-9. (c) qRT-PCR analysis was utilized to measure levels of inflammatory cytokines in different groups. (d) Protein levels of p-PI3K, PI3K, p-AKT, and AKT in MH7A and RA-FLS were evaluated by western blot analysis. *p < 0.05, **p < 0.01, ***p < 0.001 vs. control, ^#^p < 0.05, ^##^p < 0.01, ^###^p < 0.001 vs. TNF-α, ^&^p < 0.05, ^&&^p < 0.01, ^&&&^p < 0.001 vs. TNF-α + curcumin.

To explore whether curcumin regulates the PI3K/AKT pathway to regulate RA progression, we used curcumin + IGF-1 (the upstream cytokine of PI3K/AKT pathway) to culture MH7A and RA-FLS cells. The MTT and colony formation assays in Figure 4(a,b) demonstrated that compared with TNF-α + curcumin group, TNF-α + curcumin + IGF-1 group showed enhanced viability and proliferation. On the contrary, the apoptosis experiments in Figure 4(c) display that IGF-1 decreased MH7A and RA-FLS cell apoptosis. Similarly, the results in Figures 4(d) and Figure 5(a) reveal that the migration and invasion were significantly inhibited in TNF-α + curcumin compared with the TNF-α group. However, the curcumin-induced suppression in migration and invasion could be restored by IGF-1 in MH7A and RA-FLS cells. The western blot analysis in Figure 5(b) illustrates that IGF-1 rescued the curcumin-induced decrease in MMP-2 and MMP-9 levels in MH7A and RA-FLS cells. Furthermore, the qRT-PCR analysis in Figure 5(c) shows that curcumin treatment resulted in the decreased inflammation response in TNF-α-stimulated MH7A and RA-FLS, and the effect of curcumin could be partially restored by IGF-1, indicating that curcumin inhibited inflammation response in MH7A and RA-FLS cells under TNF-α condition through regulating the PI3K/AKT pathway. Figure 5(d) reveals that IGF-1 rescued the suppressive effects of curcumin on ratios of p-PI3K/PI3K and p-AKT/AKT protein levels.

Considering that the consequences caused by IGF-1 do not exclusively depend on the PI3K/AKT pathway, we used a PI3K agonist 740 Y-P and an AKT1 siRNA to assess its rescue effects on curcumin in TNF-α-stimulated RA-FLSs. All the results showed that both 740 Y-P and si-AKT1 effectively rescued the effects of curcumin in the proliferation, migration, invasion, apoptosis, and inflammatory response in TNF-α-stimulated RA-FLSs (Supplementary Figure 1 and 2). These findings revealed that curcumin regulated its biological functions in RA-FLSs by the PI3K/AKT pathway.

### 3.3. Effects of curcumin on CIA in mice

**Figure 6.**
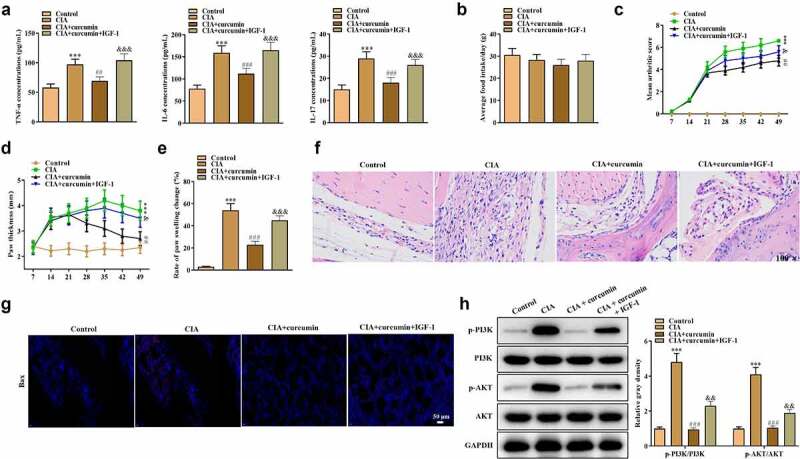
Effects of curcumin on CIA in mice. (a) Expressions of TNF-α, IL-6 and IL-17 in serum in mice of the control, CIA, CIA + curcumin, and CIA + curcumin + IGF-1 groups were detected by ELISA. (b) Food intake condition of mice. (c) Arthritis index scores in different groups. (d) Paw thickness of mice was evaluated every seven days. (e) On day 49, rate of paw swelling change was assessed. (f) H&E staining assay was performed on the swollen joints of mice from different groups. (g) Immunofluorescence staining assay was performed on the swollen joints of mice to assess expression of Bax. (h) Effects of curcumin on the PI3K/AKT signaling pathway-related proteins in mice. ***p < 0.001 vs. control, ^##^p < 0.01, ^###^p < 0.001 vs. CIA, ^&^p < 0.05, ^&&^p < 0.01, ^&&&^p < 0.001 vs. CIA + curcumin.

Effects of curcumin on RA *in vivo* were further explored using a mouse model of CIA. As shown in Figure 6(a), the concentrations of TNF-α, IL-6 and IL-17 were significantly higher in the synovial tissues in the CIA group compared with the control group, while the CII-induced increase in these proinflammatory cytokines could be partially reversed by curcumin. Moreover, IGF-1 rescued the effects of curcumin, indicating that curcumin suppressed inflammation in CIA mice. Mice in different groups showed no significant difference in average food intake (Figure 6(b)). As shown in Figure 6(c-e), compared with the control group, CIA mice showed increased arthritis index and foot swelling. Curcumin reduced arthritis index and foot swelling in CII-stimulated mice, while such effect was further rescued by IGF-1. The results of H&E staining of joint sections of mice in each group are shown in Figure 6(f). Compared with the control group, the synovial cells in the CIA group proliferated obviously with inflammatory cell infiltration, and the hyaline cartilage on the joint surface was destroyed and replaced by fibrous connective tissue with disorganized and irregular cell arrangement. Synovial proliferation was occasionally seen in the curcumin group, and there was no apparent cartilage damage, and no neovascular opacification was seen. However, effect of curcumin was further rescued by IGF-1. The western blot analysis was conducted. As shown in Figure 6(g), ratios of p-PI3K/PI3K and p-AKT/AKT were increased in the CIA group. However, the increase could be partially counteracted by treatment with curcumin, while IGF-1 treatment further rescued the effect of curcumin. These results indicated that curcumin ameliorated RA progression *in vivo* and its effects were associated with the PI3K/AKT pathway.

## 4. Discussion

Hyperproliferation and insufficient apoptosis of FLSs are usually considered to be the pathological basis of RA [19]. In addition, RA-FLSs increase the expression of MMPs, degrade the cartilage extracellular matrix, and block the supply of articular cartilage nutrients, leading to joint erosion and bone destruction [20]. In the present study, we used TNF-α-stimulated RA-FLS to mimic RA development *in vitro*. The results depicted that cell proliferation, migration, invasion, and inflammation were significantly increased in MH7A and primary RA-FLSs after TNF-α treatment. However, cell apoptosis was prominently inhibited, implying the construction of the RA in vitro model was successfully built. Curcumin inhibited the proliferation, migration, invasion, inflammation, and increased cell apoptosis in RA-FLSs, indicating that curcumin plays a protective role in RA.

Moreover, FLSs secrete a variety of chemokines, which recruit macrophages, T cells, and B cells to migrate to the joints, enhance the inflammatory response, and indirectly destroy bones and joints [21]. The anti-inflammatory effects of curcumin have been reported in previous studies, while its potential anti-inflammatory effects in RA are not yet known [22,23]. As an antioxidant, curcumin can reduce oxidative stress, protect cells from oxidative stress damage, and play an active role in preventing and treating arthritis [24]. Another research evaluated the role of curcumin in osteogenesis, which showed that curcumin could reduce bone resorption in osteoporotic rats [25]. In the present study, we found that curcumin could inhibit inflammation response in MH7A and primary RA-FLSs by reducing TNF-α, IL-6, and IL-17 levels. *In vivo* experimental results depicted that administration with curcumin could reverse the loss of appetite, increased arthritic index scores, hind paw swelling, inflammation cytokine levels, and joint destruction in a mouse model of CIA, which further supports the protective role of curcumin in RA.

The potential of curcumin to exert various biological effects is achieved by its ability to interact with multiple molecular targets or modulate multiple signaling pathways. Numerous studies have found that curcumin could stimulate several molecules of the signaling pathways, including NF-κB [26], PI3K/AKT [27], AMPK-MAPK [28], JAK/STAT [29] and Wnt/β-catenin [30]. Consistent with the previous study, in our study, we found that the core targets of curcumin for the treatment of RA included AKT1, TNF, EGFR, STAT3, and MMP9 based on bioinformatics analysis. Meanwhile, there was an excellent binding ability between curcumin and AKT1. The GO and KEGG enrichment analysis further showed that curcumin has close association with the PI3K/AKT signaling pathway.

Moreover, activation of the PI3K/AKT signaling pathway leads to aberrant expression of a variety of downstream effector molecules, exerting anti-apoptotic and pro-survival effects [31,32]. AKT activates the NF-κB pathway, which exerts a pro-inflammatory effect [33]. This study used PI3K/AKT upstream cytokine IGF-1 [34], PI3K/AKT agonist 740 Y-P, and si-AKT1 to examine whether curcumin modulates RA development by the PI3K/AKT pathway. The results showed that IGF-1, 740 Y-P, and si-AKT1 rescued the effects of curcumin on the proliferation, apoptosis, migration, invasion, and inflammation of RA-FLSs, indicating that curcumin functions on the RA-FLSs by the PI3K/AKT pathway. Moreover, curcumin rescued the CII-stimulated increase in the phosphorylated levels of PI3K and AKT, while IGF-1 further rescued the effects of curcumin on the symptoms of the CII-stimulated mice, indicating that the alleviating effects of curcumin on CII-stimulated mice were associated with the PI3K/AKT pathway.

## 5. Conclusion

Curcumin binds with AKT1 and inactivates the PI3K/AKT pathway to suppress the proliferation, migration, invasion, inflammation and to promote cell apoptosis in RA-FLSs. Furthermore, curcumin alleviates RA symptoms in a mouse model of CIA and its beneficial effects were closely associated with the PI3K/AKT pathway. These findings provide evidence for using curcumin in future clinical treatment of RA through its suppressive effect on the PI3K/AKT pathway.

## Supplementary Material

Supplemental Material

## Data Availability

All data collection and analysis were conducted under double blind conditions and were supported by the Jinling Hospital, School of Medicine, Nanjing University. We will provide the original data at any time if necessary.
