# Supplementary material for: Curcumin alleviates rheumatoid arthritis progression through the phosphatidylinositol 3-kinase/protein kinase B pathway: an in vitro and in vivo study
Source: Bioengineered. 2022 May 24;13(5):12899–911. doi: 10.1080/21655979.2022.2078942 (PMC9276000; doi:10.1080/21655979.2022.2078942)
Supplement: Supplemental Material [file KBIE_A_2078942_SM3521.zip › supplementary/ethical.pdf]

东部战区总医院动物实验伦理审查批件

批件号：2021JHDWLS-002

项目名称	姜黄素治疗类风湿关节炎的机制研究
研究单位	东部战区总医院
研究人员	徐子涵 等
审查日期	2021 年 3 月 7 日
审查地点	东部战区总医院

审查意见

经东部战区总医院实验动物伦理委员会审查，认为该方案动物实验部分符合实验动物福利伦理相关要求，同意按此方案进行。

2021 年 3 月 7 日

东部战区总医院实验动物伦理委员会

（代章）

中国人民解放军东部战区总医院
