# Supplementary figures and images for: Curcumin alleviates rheumatoid arthritis progression through the phosphatidylinositol 3-kinase/protein kinase B pathway: an in vitro and in vivo study
Source: Bioengineered. 2022 May 24;13(5):12899–911. doi: 10.1080/21655979.2022.2078942 (PMC9276000; doi:10.1080/21655979.2022.2078942)

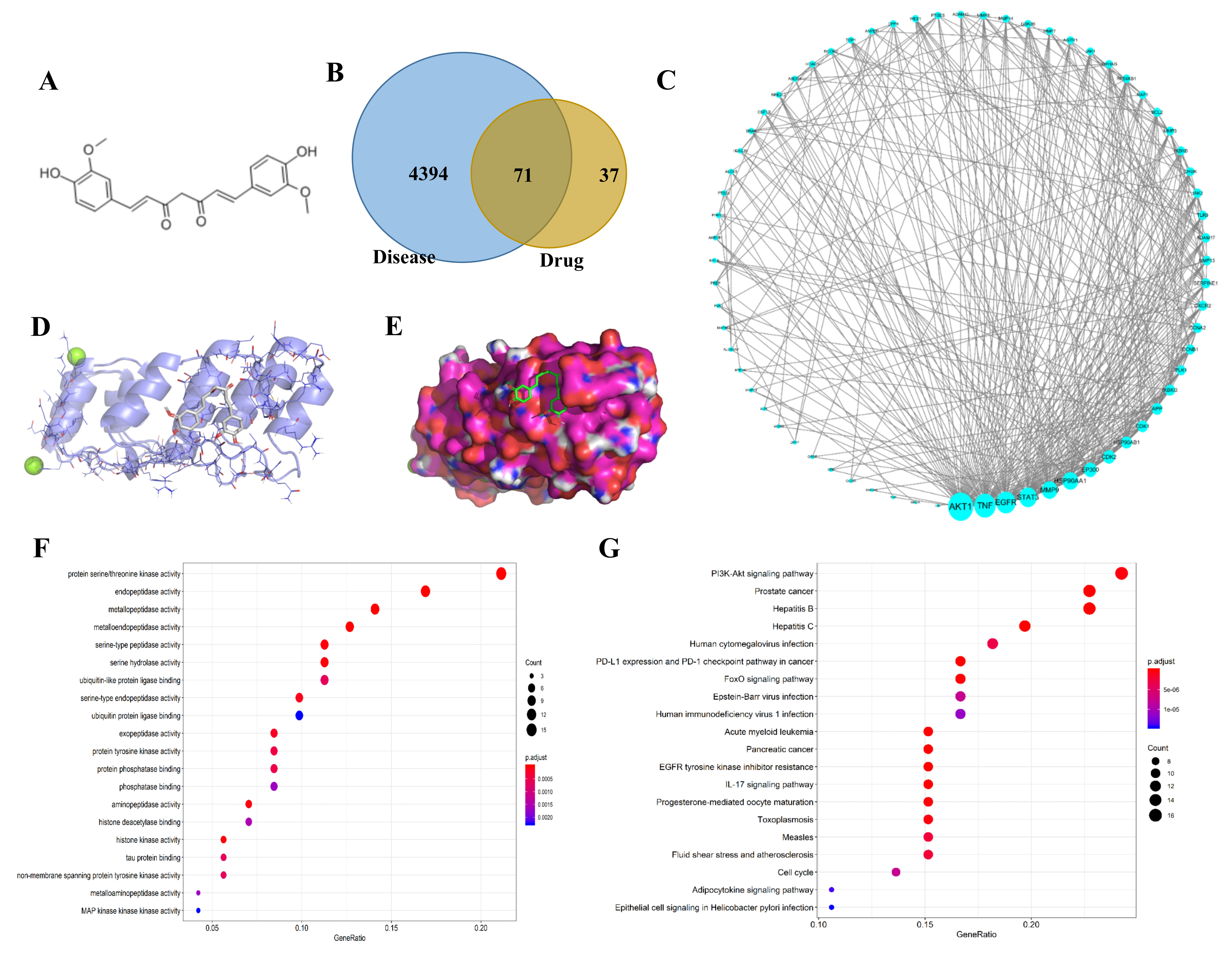

Supplement: Supplemental Material [file KBIE_A_2078942_SM3521.zip › supplementary/Supplementary Figure 1.tif]

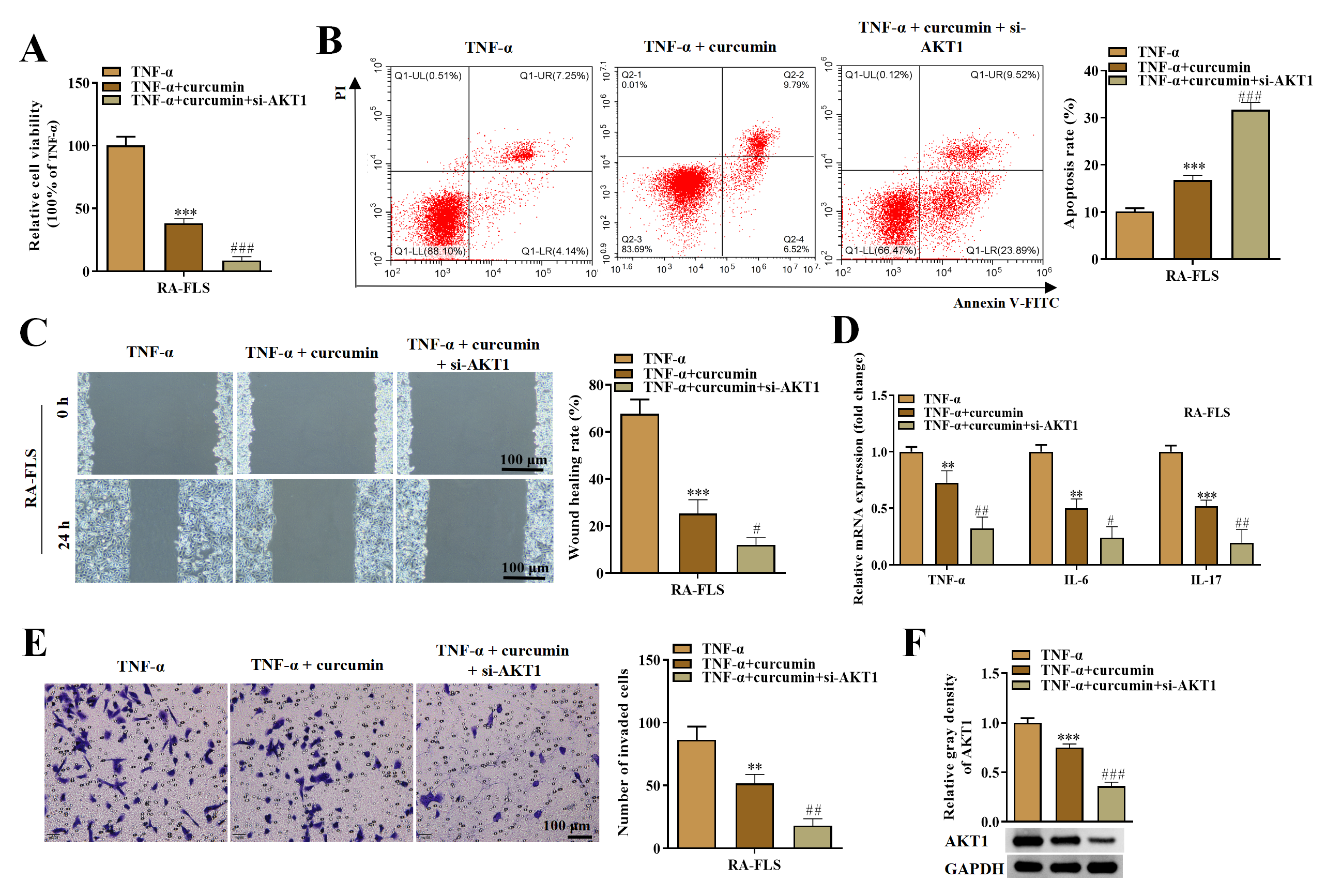

Supplement: Supplemental Material [file KBIE_A_2078942_SM3521.zip › supplementary/Supplementary Figure 2.tif]
